# UBE2I promotes metastasis and correlates with poor prognosis in hepatocellular carcinoma

**DOI:** 10.1186/s12935-020-01311-x

**Published:** 2020-06-12

**Authors:** Hao Yang, Shan Gao, Jing Chen, Weiyang Lou

**Affiliations:** 1 Clinical Research Institute, Zhejiang Provincial People’s Hospital, People’s Hospital of Hangzhou Medical Hospital, Hangzhou, 310014 Zhejiang China; 2 Department of Anus & Intestine Surgery, Zhejiang Provincial People’s Hospital, Hangzhou, 310014 Zhejiang China; 3 Department of Oncology, First Affiliated Hospital of Jiaxing University, Jiaxing, 314000 Zhejiang China; 4 Department of Breast Surgery, First Affiliated Hospital, College of Medicine, Zhejiang University, Hangzhou, 310003 Zhejiang China

**Keywords:** UBE2I, Hepatocellular carcinoma, Diagnosis, Prognosis, Migration, Invasion, Metastasis, microRNA (miRNA), Protein–protein interaction (PPI)

## Abstract

**Background:**

A comprehensive investigation of ubiquitin-conjugating enzyme E2I (UBE2I) in cancer is still insufficiency. In this study, we aimed to analyze its role and mechanism in cancer by combination of bioinformatic analysis and experimental validation.

**Methods:**

The expression profile of UBE2I in human cancers were obtained using GEPIA. Kaplan–Meier plotter was used to assess the prognostic values of UBE2I in diverse types of cancer. ROC curve analysis was employed to determine the diagnostic role of UBE2I in hepatocellular carcinoma (HCC). The expression differences based on various clinicopathological features was evaluated by UALCAN. Wound healing assay and transwell invasion assay were used to detected the effects of UBE2I on migration and invasion of HCC cells, respectively. The miRNA regulatory mechanism of UBE2I was successively investigated by binding prediction, expression analysis, survival analysis and dual-luciferase reporter assay. The correlation of UBE2I mRNA expression and UBE2I promoter methylation level was assessed using cBioPortal. STRING was finally introduced to perform co-expression analysis and enrichment analysis for UBE2I.

**Results:**

UBE2I was upregulated in HCC, correlated with cancer progression and poor prognosis of HCC. We also found a significant diagnostic value of UBE2I in HCC. Functional experiments revealed that knockdown of UBE2I significantly inhibited HCC migration and invasion. Further research on mechanism suggested that loss of inhibition of hsa-miR-195-3p and dysregulation of UBE2I promoter methylation might account for UBE2I overexpression in HCC. Analysis of UBE2I-invovled regulatory network identified six key genes (NSMCE2, SAE1, UBA2, RANGAP1, SUMO1 and SUMO2) whose expression linked to poor prognosis in HCC.

**Conclusions:**

In conclusion, UBE2I may be a promising therapeutic target and biomarker in cancer, especially HCC.

## Background

As is known to all, ubiquitin–proteasome pathway (UPP), consisting of ubiquitin, ubiquitin-activating enzyme (E1), ubiquitin-conjugating enzyme (E2), ubiquitin-ligating enzyme (E3) and proteasome, plays important roles in post-translationally regulating protein expression, thus participating in almost all life activities of eukaryotes [1, 2]. Previous studies have reported that dysregulation of UPP is linked to development of multiple human disorders, such as systemic inflammation [3, 4] and cancer [5–7].

In 1996, Watanabe et al. identified a novel cell cycle-associated gene *UBC9*, containing an open reading frame of 474 nucleotides encoding 158 amino acids [8]. This gene encodes Ubiquitin-conjugating enzyme E2I (UBE2I), which is a member of E2 enzyme family. UBE2I is a key regulator of body immunity. For example, the team of Wang A indicated that UBI2I was required for positive selection and late-stage maturation of thymocytes [9]. UBE2I is also found to favorably impact cardiac function in compromised hearts by mediating SUMOylation [10]. Moreover, several reports have shown that abnormal expression of UBE2I results in occurrence and progression of multiple human cancers, including breast cancer [11, 12], glioma [13, 14], lung cancer [15], head and neck squamous cell carcinoma [16], osteosarcoma [17] and hepatocellular carcinoma [18].

However, to date, a comprehensive analysis of UBE2I in human cancers remains absent. Elucidation of UBE2I expression, function and mechanism in human cancers may provide an effective therapeutic target and a promising biomarker for diagnosing or predicting prognosis of cancer patients. In the study, expression profile of UBE2I in 33 various cancers was first analyzed using data from the Cancer Genome Atlas (TCGA) and the Genotype-Tissue Expression (GTEx) projects. Then, HCC was selected as the studied cancer of interest by combination of the various stage expression analysis and survival analysis of UBE2I. Further investigation revealed that UBE2I expression correlated with progression of HCC. Moreover, ROC curve analysis showed a significant diagnostic value of UBE2I for HCC patients. Then, two potential dysregulated mechanisms of UBE2I in HCC were explored. We also identified several positively correlated genes that are involved in UBE2I-associated modulatory network. Finally, survival analysis and enrichment analysis for these genes were performed.

## Materials and methods

### GEPIA database analysis

GEPIA, a newly developed interactive web server for analyzing the RNA sequencing data of 9736 tumor samples and 8587 normal samples from the TCGA and GTEx projects, provides a variety of functions, including tumor/normal differential expression analysis, profiling according to cancer types or pathological stages and correlation analysis [19]. In this study, GEPIA database was first employed to analyze UBE2I expression profiling in 33 different cancer types and determine expression differences of UBE2I among various major stages in cancers. Moreover, GEPIA database was also introduced to assess the expression relationships between two genes in HCC. *P* value < 0.05 was considered as statistically significant.

### Kaplan–Meier plotter analysis

The prognostic values of UBE2I in kidney renal clear cell carcinoma (KIRC), liver hepatocellular carcinoma (LIHC, HCC) and stomach adenocarcinoma (STAD) were evaluated using Kaplan–Meier plotter as previously described [20, 21]. Briefly, UBE2I was first entered into the database. Subsequently, Kaplan–Meier survival plots were automatically generated, and statistical analytic results, including hazards ratio (HR), 95% confidence interval (CI) and logrank P-value, were presented on the webpage. This database was also used to perform survival analysis of miRNAs and other genes in HCC. Logrank P-value < 0.05 was considered as statistically significant.

### Oncomine database analysis

Oncomine database is a cancer microarray database and integrated data-mining platform [22]. Oncomine database was utilized to analyze UBE2I expression in HCC. P-value < 0.05, |fold change (FC)| > 1.5 and gene rank in the top 10% were set as the thresholds for choosing datasets of interest.

### UALCAN database analysis

UALCAN, an online web server for facilitating tumor subgroup gene expression and survival analyses based on the TCGA data, was used to detect expression levels of UBE2I according to several different clinicopathological features, containing nodal metastasis, individual cancer stage and tumor grade [23]. P-value < 0.05 was considered as statistically significant.

### ROC curve analysis

The diagnostic role of UBE2I in HCC was assessed by receptor operating characteristic (ROC) curve analysis based on TCGA normal liver samples and HCC samples. P-value < 0.05 was considered as statistically significant.

### miRNet database analysis

The potential miRNAs binding to UBE2I were predicted through miRNet, which is a comprehensive database and analytic platform to explore miRNA-target interactions and functional associations by network-based visual analysis [24, 25]. The miRNA-UBE2I interactions were re-entered into Cytoscape software (Version 3.6.0) to establish miRNA-UBE2I regulatory network.

### starBase database analysis

The expression levels of potential miRNAs in HCC were determined by starBase database, which is a web server for decoding miRNA-mRNA interaction maps from large-scale CLIP-Seq data [26, 27]. starBase database was also employed to evaluate the expression correlation of miRNA and UBE2I in HCC. P-value < 0.05 was considered as statistically significant.

### cBioPortal database analysis

cBioPortal is a database used to explore, visualize and analyze multidimensional cancer genomics data [28]. cBioPortal was utilized to assess the association between UBE2I promoter methylation level and UBE2I mRNA expression in HCC. P-value < 0.05 was considered as statistically significant.

### STRING database analysis

STRING database (https://string-db.org/) was introduced to probe the UBE2I-involved protein–protein interaction (PPI) network. The PPI network was directly downloaded from the webpage. Only these interactions with score ≥ 0.4 were included. Gene Ontology (GO) functional annotation and pathway enrichment analysis were also performed by this database.

### Cell lines and cell culture

Two human HCC cell lines, HepG2 and Bel7402, were purchased from the Institute of Biochemistry and Cell Biology of the Chinese Academy of Sciences (Shanghai, China). These cells were maintained in Dulbecco’s modified Eagle’s medium (DMEM; Gibco) supplemented with 10% fetal bovine serum (FBS; Biological Industries) under a humidified atmosphere of 5% CO_2_ at 37 °C.

### Cell transfection

Small interfering RNA (siRNA) targeting UBE2I (si-UBE2I) and negative control (NC) siRNA (si-NC) were designed and synthesized by Guangzhou Ribobio Co. Ltd. (Guangzhou, China). miR-195-3p mimic and mimic negative control (mimic-NC) were also obtained from Guangzhou Ribobio Co. Ltd. (Guangzhou, China). Cells were seeded into six-well plates, after which transfection was introduced by Lipofectamine™ 3000 (Invitrogen, Shanghai, China) according to the manufacturer’s instruction.

### RNA isolation and qPCR

RNA isolation and qPCR were performed as previously described [29, 30].

### Wound healing assay

Firstly, pre-transfected cells were plated into six-well plates. After these cells were grown to 100% confluence, a micropipette tip was used to make a cross wound. Subsequently, photographs were taken using at 0 h and 24 h post-transfection.

### Transwell assay

Transwell invasion assay was employed to determine the invaded ability of HCC cells. Firstly, transwell inserts (Corning, USA) were coated with Matrigel (BD Bioscience, USA). Subsequently, 50,000 cells were suspended into 0.2 mL serum-free medium and then added into the pre-coated inserts. 0.6 mL medium containing 10% FBS were added to the lower compartment. After culturing for 48 h at 37 °C, the cells on the super surface of the membrane were removed using a cotton swab and the cells on the lower surface of the membrane were successively fixed with 10% methanol and stained with 0.1% crystal violet. Finally, five fields of each insert were randomly selected through a microscopy.

### Dual-luciferase reporter assay

The 3′-UTR fragments of UBE2I containing the wild-type or mut-type miR-195-3p-binding site were constructed and cloned into psi-CHECK2 luciferase reporter vector (Promega, USA). HepG2 cells were co-transfected with luciferase plasmids and miR-195-3p mimics. 48 h post-transfection, luciferase activity was measured with the Dual-Luciferase Reporter Assay System (Promega, USA) and firefly luciferase activity was normalized to Renilla luciferase activity.

### Statistical analysis

The statistical analysis of in silico analyses has been performed by the online bioinformatic tools as mentioned above. The diagnostic value of UBE2I in HCC was determined by ROC curve analysis using GraphPad Prism software (Version 7). Differences between two groups of data and statistical significance were analyzed by Students’ *t*-test. P-value < 0.05 was considered to indicate a statistically significant difference.

## Results

### Expression profile and prognostic values of UBE2I in human cancers

**Fig. 1:**
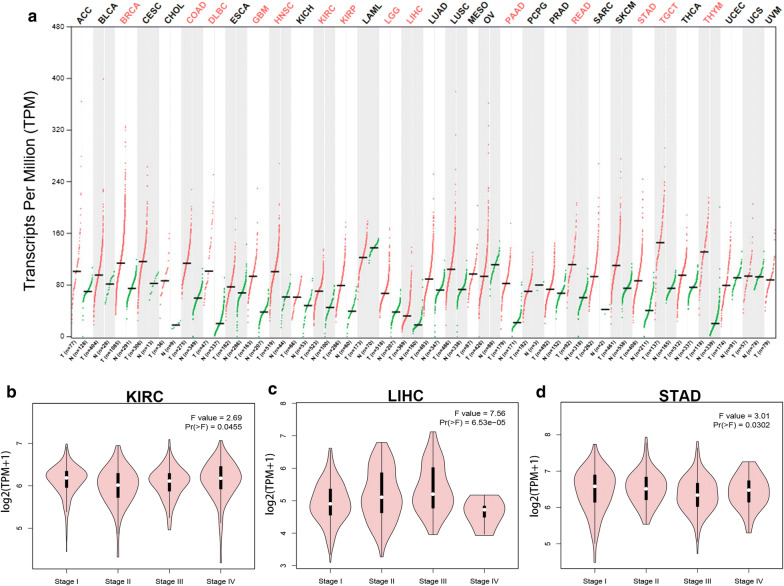
Expression of UBE2I in human cancers determined by GEPIA database. **a** The expression profile of UBE2I in 33 human cancers. **b** The expression difference of UBE2I among various major stage of KIRC. **c** The expression difference of UBE2I among various major stage of LIHC. **d** The expression difference of UBE2I among various major stage of STAD

**Fig. 2:**
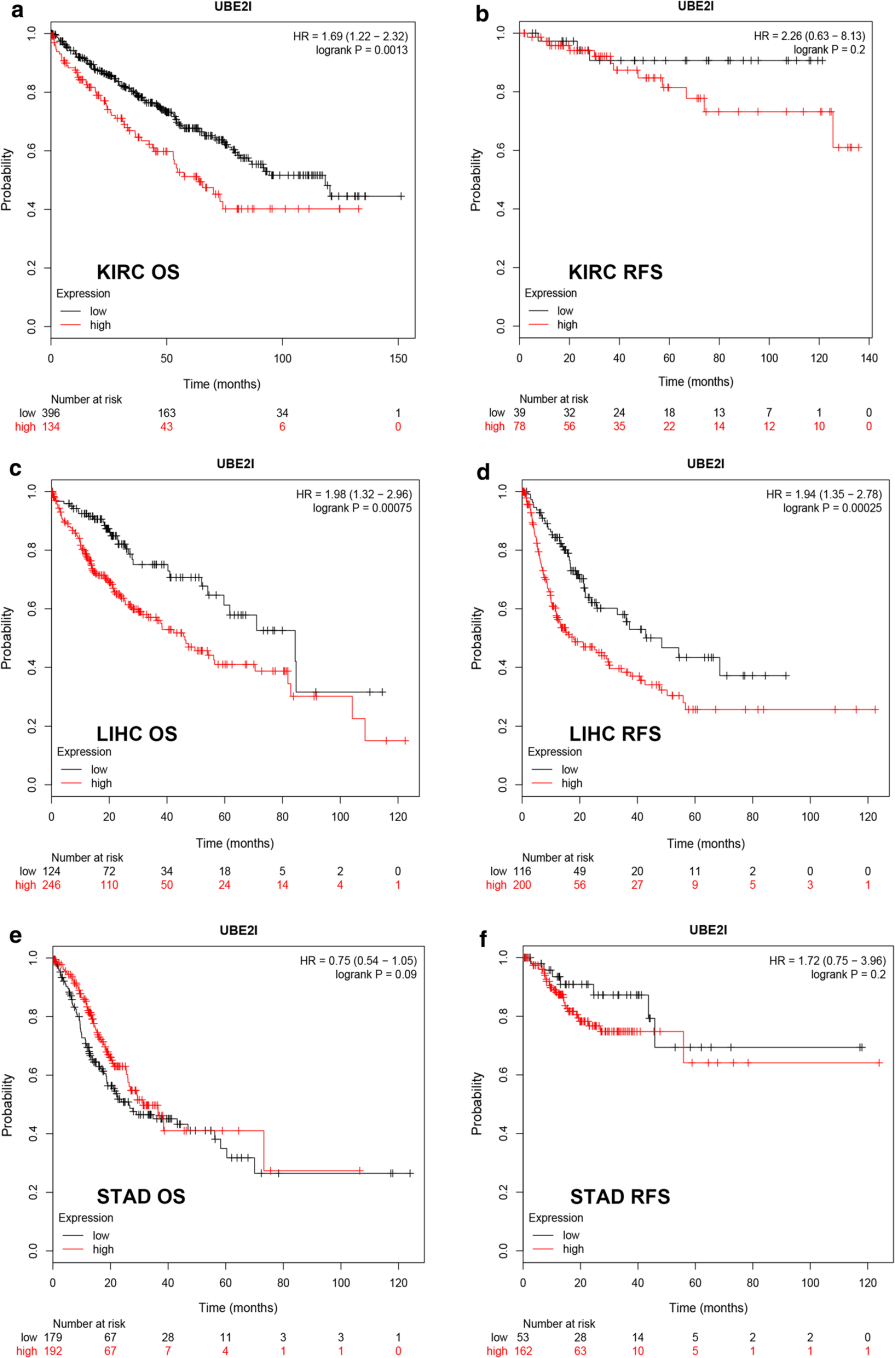
Survival analysis of UBE2I in KIRC, LIHC and STAD determined by Kaplan–Meier plotter database. **a** The prognostic value (OS) of UBE2I in KIRC. **b** The prognostic value (RFS) of UBE2I in KIRC. **c** The prognostic value (OS) of UBE2I in LIHC. **d** The prognostic value (RFS) of UBE2I in LIHC. **e** The prognostic value (OS) of UBE2I in STAD. **f** The prognostic value (RFS) of UBE2I in STAD. Note: OS, overall survival; RFS, relapse free survival. Logrank P-value < 0.05 was considered as statistically significant

The data from GTEx data and TCGA normal tissues and cancer tissues revealed that UBE2I was markedly overexpressed in 13 types of cancer (BRCA, COAD, DLBC, GBM, HNSC, KIRC, KIRP, LGG, LIHC, PAAD, READ, STAD, TGCT and THYM) when compared with corresponding normal controls (Fig. 1a). Next, we determined the expression differences of UBE2I among various major stages in the 13 human cancers using GEPIA database. The results demonstrated that expression of UBE2I among different major stages presented statistically difference only in 3 cancer types, containing KIRC (Fig. 1b), LIHC (Fig. 1c) and STAD (Fig. 1d). Therefore, KIRC, LIHC and STAD were selected as cancer candidates for further investigation. Subsequently, we performed survival analysis of UBE2I in KIRC, LIHC and STAD using Kaplan–Meier plotter database. Two prognostic indices, overall survival (OS) and relapse free survival (RFS), were included. As shown in Fig. 2a, high expression of UBE2I was significantly associated with unfavorable OS of KIRC. No significant association of UBE2I expression and RFS of KIRC was observed (Fig. 2b). As presented in Fig. 2c, d, LIHC patients with higher expression had poorer OS and RFS, respectively. For STAD, UBE2I expression was not significantly correlated with patients’ OS and RFS as shown in Fig. 2e, f, respectively. The results from expression analysis and survival analysis together suggest that LIHC may be the most suitable cancer type for UBE2I study.

### Diagnostic role of UBE2I in HCC

**Fig. 3:**
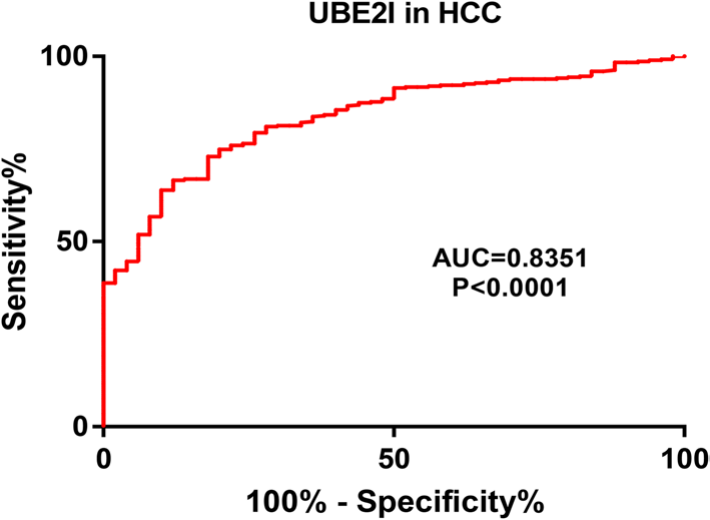
The diagnostic value of UBE2I in HCC based on the TCGA normal liver and HCC data. P-value < 0.05 was considered as statistically significant

In view of the prognostic value of UBE2I in HCC, in this part, we further evaluated the diagnostic role of UBE2I for patients with HCC by performing ROC curve analysis using TCGA normal liver and HCC data. Figure 3 revealed that UBE2C possessed a significant diagnostic ability distinguishing HCC from normal controls, with area under curve (AUC) equal to 0.8351. The current result indicates that UBE2I may be a promising diagnostic biomarker for patients with HCC.

### UBE2I is highly expressed in HCC and correlates with progression of HCC

**Fig. 4:**
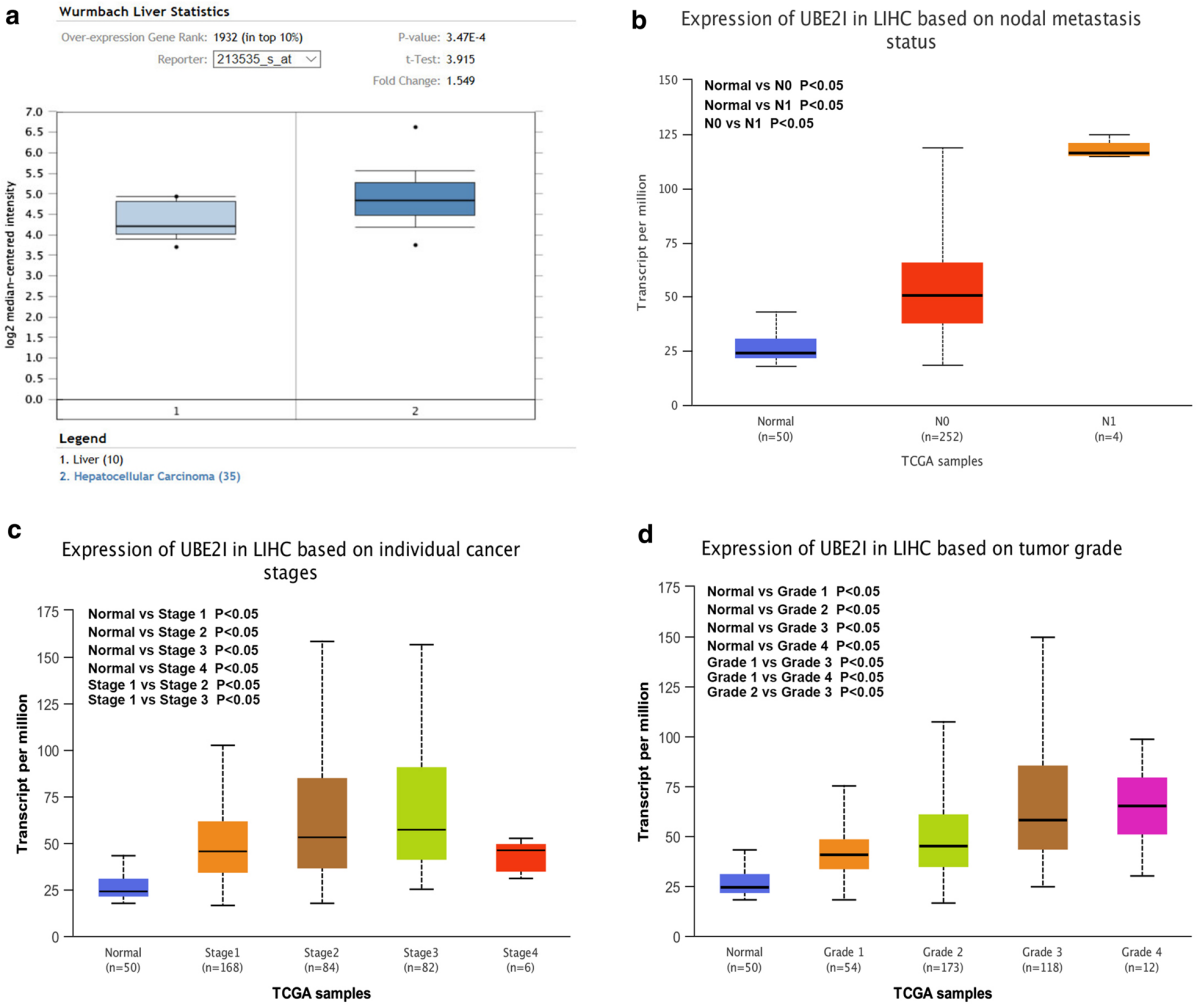
UBE2I expression in HCC based on nodal metastasis status, individual cancer stage or tumor grade. **a** The expression level of UBE2I in HCC samples compared with normal liver samples determined by Oncomine database. **b** UBE2I expression in HCC based on nodal metastasis status. **c** UBE2I expression in HCC based on individual cancer stage. **d** UBE2I expression in HCC based on tumor grade. **e** UBE2I protein expression levels in HCC cancer tissue and normal tissue were analyzed using immunohistochemical staining from HumanProteinAtlas database. P-value < 0.05 was considered as statistically significant

To further validate the high expression of UBE2I in HCC, Oncomine database was employed. As presented in Fig. 4a, we also found that UBE2I was obviously overexpressed in HCC tissues when compared with normal liver tissues. Moreover, the analytic result from UALCAN database demonstrated that HCC patients with nodal metastasis had statistically higher expression of UBE2I than that in HCC patients without nodal metastasis (Fig. 4b). As shown in Fig. 4c, d, UBE2I expression was upregulated in advanced stage HCC and poorly differentiated grade HCC compared with early stage HCC and well differentiated grade HCC in general, respectively. Moreover, UBE2I protein expression level was also increased in HCC cancer tissue when compared with normal tissue (Fig. 4e). All these findings further confirm that UBE2I is highly expressed in HCC and correlates with HCC progression.

### Knockdown of UBE2I inhibits HCC invasion and migration

**Fig. 5:**
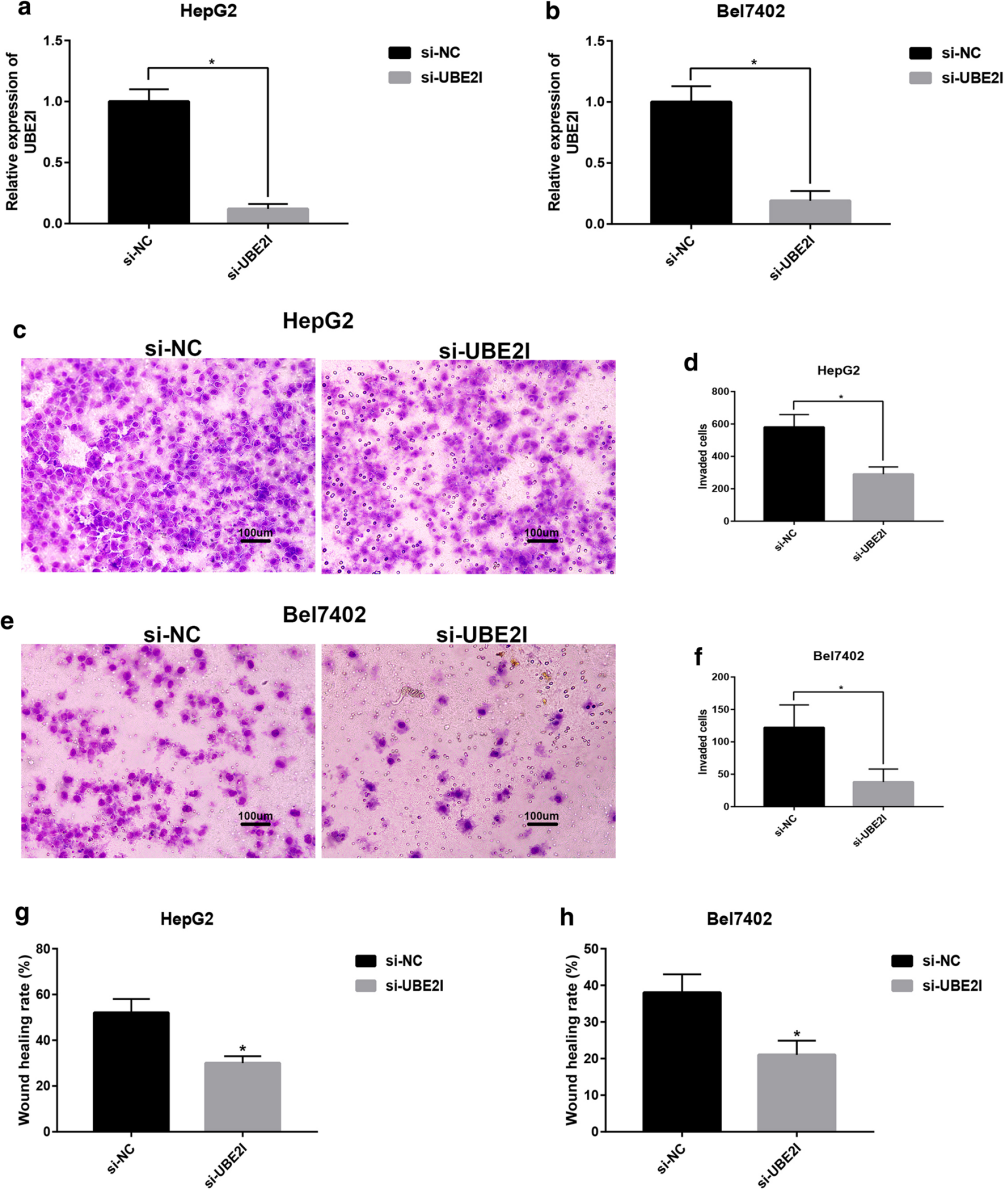
Knockdown of UBE2I inhibited invasion and migration of HCC cells. **a** The knockdown effect of si-UBE2I in HepG2 cells. **b** The knockdown effect of si-UBE2I in Bel7402 cells. **c** Treatment of si-UBE2I significantly suppressed HepG2 invasion. **d** The quantitative analysis of the effect of si-UBE2I on HepG2 invasion. **e** Treatment of si-UBE2I significantly suppressed Bel7402 invasion. **f** The quantitative analysis of the effect of si-UBE2I on Bel7402 invasion. **g** Treatment of si-UBE2I significantly suppressed HepG2 migration. **h** Treatment of si-UBE2I significantly suppressed Bel7402 migration. Bar scale: 100 um. P-value < 0.05 was considered as statistically significant

As UBE2I expression linked to nodal metastasis, advanced clinical stage, high tumor grade and poor prognosis, we supposed that UBE2I might be involved in regulation of HCC metastasis. In this part, transwell invasion assay and wound healing migration assay were performed. As shown in Fig. 5a and b, treatment of si-UBE2I in HepG2 and Bel7402 cells, the UBE2I expression was significantly decreased. Figure 5c, d revealed that knockdown of UBE2I marked reduced the invaded ability of HepG2. The similar result was also observed in LM3 as presented in Fig. 5e and f. Moreover, HepG2 and LM3 cell migration was significantly inhibited in si-UBE2I-treated groups compared with si-NC-treated groups. Taken together, UBE2I may be one HCC metastasis promoting molecule.

### The upstream mechanisms of UBE2I in HCC

**Fig. 6:**
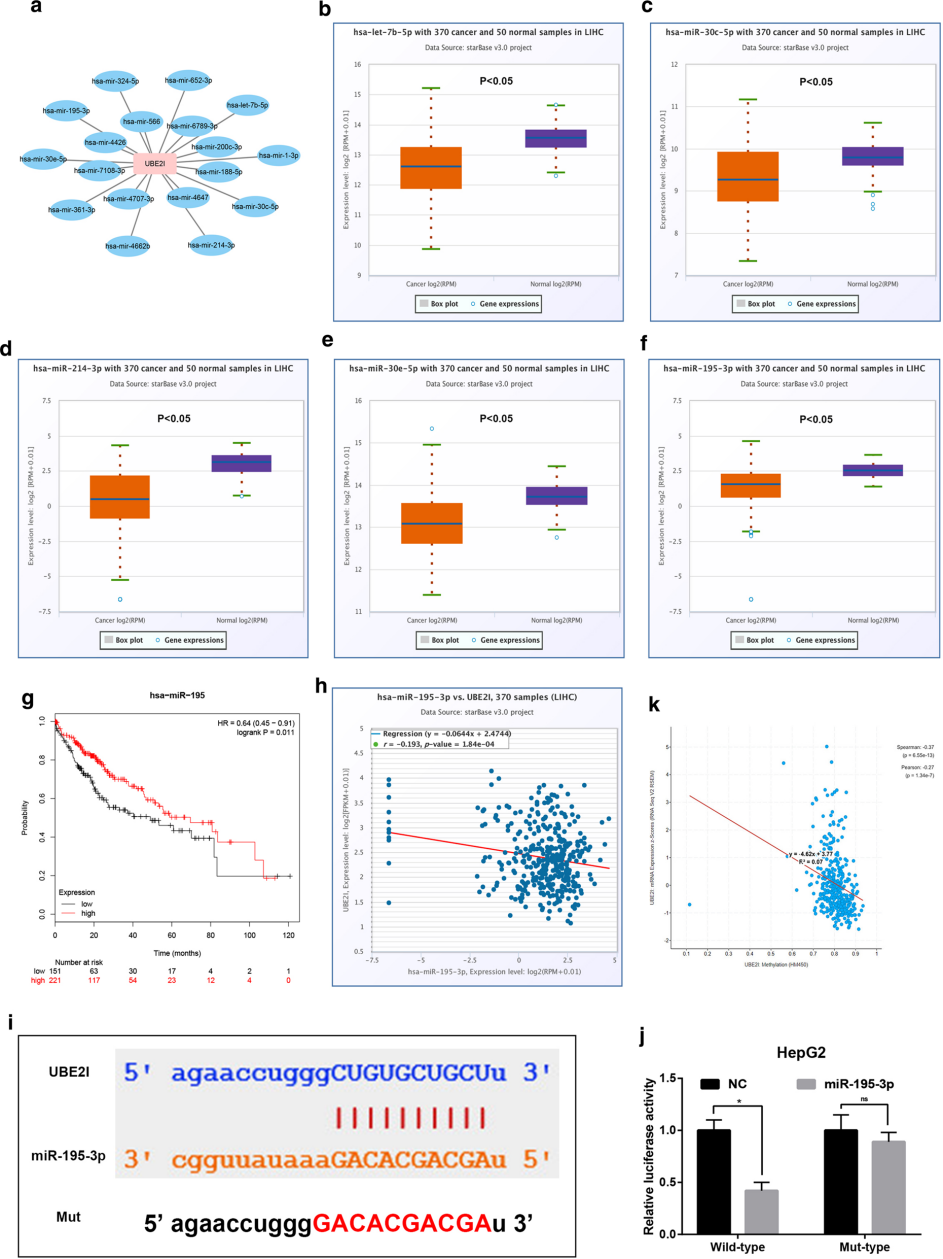
The dysregulated mechanisms of UBE2I in HCC. **a** The potential miRNAs-UBE2I regulatory network established by Cytoscape software (Version 3.6.0). **b** The expression level of hsa-let-7b-5p in HCC determined by starBase database. **c** The expression level of hsa-miR-30c-5p in HCC determined by starBase database. **d** The expression level of hsa-miR-214-3p in HCC determined by starBase database. **e** The expression level of hsa-miR-30e-5p in HCC determined by starBase database. **f** The expression level of hsa-miR-195-3p in HCC determined by starBase database. P-value < 0.05 was considered as statistically significant. **g** The prognostic value of has-miR-195-3p in HCC determined by Kaplan–Meier plotter. Logrank P-value < 0.05 was considered as statistically significant. **h** The expression correlation between UBE2I and hsa-miR-195-3p in HCC determined by starBase database. **i** The relationship between UBE2I promoter methylation level and UBE2I mRNA expression in HCC determined by cBioPortal database. **j** The binding sites of has-miR-193-5p and UBE2I was predicted by starBase and the mutant binding sequences for miR-193-5p in the 3′-UTR of UBE2I were also shown. **k** Luciferase activity was detected in HepG2 by co-transfected with a reporter plasmid carrying the wild-type or mut-type UBE2I 3′-UTR and either the miR-195-3p mimics or miR-NC. P-value < 0.05 was considered as statistically significant. “ns” represented no significant

MiRNAs are found to play important roles in cancer development and progression, mainly by targeting gene expression. Thus, we predicted the upstream binding miRNAs of UBE2I using miRNet database. As shown in Fig. 6a, 18 miRNAs were forecasted to potentially bind to UBE2I. Based on the action mechanism of miRNAs and the oncogenic role of UBE2I in HCC, the potential regulatory miRNAs of UBE2I should be tumor suppressive miRNAs in HCC. By expression analysis, we found that only hsa-let-7b-5p (Fig. 6b), hsa-miR-30c-5p (Fig. 6c), hsa-miR-214-3p (Fig. 6d), hsa-miR-30e-5p (Fig. 6e) and hsa-miR-195-3p (Fig. 6f) were significantly downregulated in HCC samples when compared with normal liver samples. Next, survival analysis for the 5 miRNAs revealed that only high expression of hsa-miR-195-3p indicated favorable prognosis of HCC as presented in Fig. 6g. Moreover, hsa-miR-195-3p expression was significantly inversely linked to UBE2I expression in HCC determined by starBase database (Fig. 6h). Figure 6i showed the potential binding sites of miR-195-3p and UBE2I. Dual-luciferase reporter assay suggested that miR-195-3p could directly bind to UBE2I (Fig. 6j). Altogether, hsa-miR-195-3p might be a critical negative modulator of UBE2I in HCC. Gene promoter methylation deregulation may cause silence or activation of tumor suppressors or oncogenes, thereby resulting in development of human cancers, including HCC. In this work, we wanted to ascertain if dysregulation of UBE2I promoter methylation is associated with UBE2I overexpression in HCC. The result from Fig. 6k demonstrated that promoter methylation level of UBE2I was significantly negatively correlated with UBE2I mRNA expression.

### UBE2I-involved PPI network

**Fig. 7:**
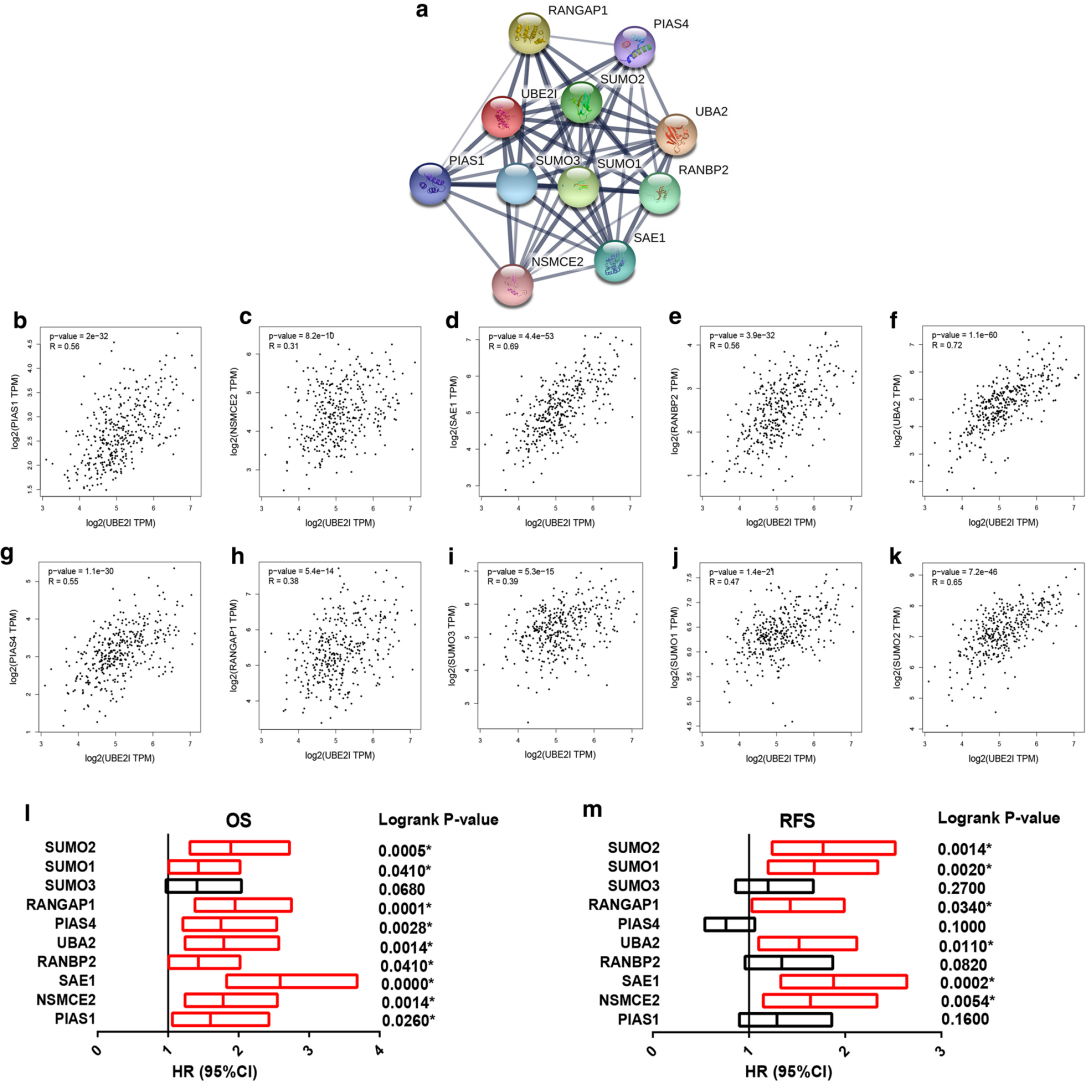
Expression correlation analysis and survival analysis of UBE2I with genes in the UBE2I-involved protein–protein interaction network. **a** The UBE2I-involved protein–protein interaction network constructed by STRING database. **b** The expression relationship between UBE2I and PIAS1 in HCC assessed by GEPIA database. **c** The expression relationship between UBE2I and NSMCE2 in HCC assessed by GEPIA database. **d** The expression relationship between UBE2I and SAE1 in HCC assessed by GEPIA database. **e** The expression relationship between UBE2I and RANBP2 in HCC assessed by GEPIA database. **f** The expression relationship between UBE2I and UBA2 in HCC assessed by GEPIA database. **g** The expression relationship between UBE2I and PIAS4 in HCC assessed by GEPIA database. **h** The expression relationship between UBE2I and RANGAP1 in HCC assessed by GEPIA database. **i** The expression relationship between UBE2I and SUMO3 in HCC assessed by GEPIA database. **j** The expression relationship between UBE2I and SUMO1 in HCC assessed by GEPIA database. **k** The expression relationship between UBE2I and SUMO2 in HCC assessed by GEPIA database. P-value < 0.05 was considered as statistically significant. **l** The prognostic values (OS, overall survival) of the 10 correlated genes of UBE2I in HCC determined by Kaplan–Meier plotter. **m** The prognostic values (RFS, relapse free survival) of the 10 correlated genes of UBE2I in HCC determined by Kaplan–Meier plotter. Red bars indicated unfavorable prognosis; black represented no statistical significance. Logrank P-value < 0.05 was considered as statistically significant

To better understand the molecular action mechanism of UBE2I, we constructed a UBE2I-involved PPI network using STRING database as shown in Fig. 7a. Three GO categories, containing biological process (BP), molecular function (MF) and cellular component (CC), were included for GO functional annotation. As listed in Table 1, the top 5 enriched GO items included protein sumoylation, positive regulation of protein sumoylation in the BP category; SUMO transferase activity, small protein activating enzyme binding and enzyme binding in the MF category; and PML body, SUMO activating enzyme complex and nuclear part in the CC category. For pathway enrichment, KEGG’s cell signaling pathway and Reactome’s cell signaling pathway were analyzed. Table 2 suggested that the proteins from UBE2I-invovled PPI network significantly enriched in ubiquitin mediated proteolysis and SUMOylation pathways. Besides, we also assessed the mRNA expression correlation of UBE2I and individual protein-coding gene from UBE2I-involved PPI network using GEPIA database. The result revealed that UBE2I expression was statistically positively linked to PIAS1 (Fig. 7b), NSMCE2 (Fig. 7c), SAE1 (Fig. 7d), RANBP2 (Fig. 7e), UBA2 (Fig. 7f), PIAS4 (Fig. 7g), RANGAP1 (Fig. 7h), SUMO3 (Fig. 7i), SUMO1 (Fig. 7j) or SUMO2 (Fig. 7k) expression in HCC. Survival analysis showed that most of the 10 correlated genes were linked to poor OS and RFS as shown in Fig. 7l and m, respectively. Notably, high expression of NSMCE2, SAE1, UBA2, RANGAP1, SUMO1 and SUMO2 indicated unfavorable OS and RFS in HCC.

**Table 1:** The top 5 GO items related to proteins involved in UBE2I network

GO-term	Description	False discovery rate
Biological process (BP)		
GO:0016925	Protein sumoylation	8.43e−24
GO:0033235	Positive regulation of protein sumoylation	5.45e−06
GO:0032436	Positive regulation of proteasomal ubiquitin-dependent protein catabolic process	9.15e−06
GO:0043085	Positive regulation of catalytic activity	8.30e−04
GO:0065009	Regulation of molecular function	1.20e−03
Molecular function (MF)		
GO:0019789	SUMO transferase activity	9.18e−14
GO:0044388	Small protein activating enzyme binding	2.99e−10
GO:0019899	Enzyme binding	9.43e−08
GO:0031386	Protein tag	3.48e−07
GO:0008022	Protein C-terminus binding	6.65e−07
Cellular component (CC)		
GO:0016605	PML body	3.45e−12
GO:0031510	SUMO activating enzyme complex	1.34e−07
GO:0044428	Nuclear part	1.73e−06
GO:0005654	Nucleoplasm	5.48e−06
GO:0044614	Nuclear pore cytoplasmic filaments	5.48e−05

**Table 2:** The top 5 KEGG and Reactome pathways related to proteins involved in UBE2I network

Pathway-term	Description	False discovery rate
KEGG pathways		
hsa04120	Ubiquitin mediated proteolysis	5.25e−08
hsa03013	RNA transport	6.03e−08
hsa05418	Fluid shear stress and atherosclerosis	1.20e−04
hsa04064	NF-kappa B signaling pathway	2.20e−03
hsa04630	Jak-STAT signaling pathway	5.00e−03
Reactome pathways		
HSA-2990846	SUMOylation	9.18e−14
HSA-3108232	SUMO E3 ligases SUMOylate target proteins	2.99e−10
HSA-3108214	SUMOylation of DNA damage response and repair proteins	9.43e−08
HSA-3065678	SUMO is transferred from E1 to E2 (UBE2I, UBC9)	3.48e−07
HSA-4615885	SUMOylation of DNA replication proteins	6.65e−07

## Discussion

UBE2I is a member of the E2 enzyme family, involving in post-translationally regulating protein expression. A variety of investigations have suggested that UBE2I plays key roles in occurrence and development of several cancers. However, to the best of our knowledge, a study regarding its expression, functions and mechanisms in cancer remains absent. Thus, this study was conducted.

Our study conducted an analysis of UBE2I in 33 cancers using the data from TCGA and GTEx databases. The analytic result provided evidences that UBE2I was upregulated in 13 cancers and may act as an oncogene in these cancers. To find the studied cancer of interest, we assessed expression differences of UBE2I among various major stages in the 13 cancers. Only three cancers, KIRC, HCC and STAD, possessed statistical differences. Subsequently, the prognostic values (OS and RFS) of UBE2I in the three cancers were evaluated using Kaplan–Meier plotter database. High expression of UBE2I was significantly negatively correlated with both OS and RFS only in HCC. Fang et al. found that UBE2I was markedly overexpressed in HCC cell lines and clinical samples compared with normal controls [18]. They also indicated that UBE2I decreased the sensitivity of HCC to doxorubicin. This report together with our analytic results suggest that UBE2I may be a critical oncogene in HCC and may be developed as a promising therapeutic target in the future. We also found that UBE2I had a significant diagnostic value in HCC by ROC curve analysis. All these findings further imply that UBE2I may be an oncogene in HCC and may be a potential diagnostic biomarker for HCC patients.

For further validating UBE2I expression in HCC, Oncomine database was employed. The analytic result revealed that UBE2I was upregulated in HCC, partially supporting the accuracy of our previous bioinformatic analysis. Subsequently, we determined the expression level of UBE2I in HCC based on some clinicopathological features, including nodal metastasis, individual cancer stage and tumor grade. In general, UBE2I was correlated with development and progression of HCC. Functional experiments suggested that inhibition of UBE2I significantly reduced the invaded and migrated abilities of HCC, further supporting the oncogenic roles of UBE2I in HCC.

Next, we explored possible mechanisms causing UBE2I upregulation in HCC. It has been widely acknowledged that miRNAs are involved in negatively regulating downstream target gene expression [29–32]. Furthermore, in 2012, Zhao et al. showed that UBE2I expression was directly targeted by hsa-miR-214-3p in glioma [13]. Therefore, we predicted the upstream miRNAs of UBE2I by miRNet, which is a comprehensive database for miRNA-associated studies [24, 25]. Finally, 18 potential miRNAs were identified. Then, the expression levels and prognostic roles of these miRNAs were determined using TCGA data by starBase and Kaplan–Meier plotter, respectively. The results revealed that hsa-miR-195-3p was the most potential regulatory miRNA of UBE2I, with both downregulation in HCC compared with normal liver and possessing favorable prognosis in HCC patients with high expression UBE2I. Moreover, an inverse expression association between hsa-miR-195-3p and UBE2I was observed in HCC. Additionally, for the first time, we demonstrated that the promoter methylation level of UBE2I was significantly negatively linked to UBE2I mRNA expression in HCC. Taken together, loss of inhibition of hsa-miR-195-3p and dysregulation of promoter methylation level of UBE2I may account for UBE2I overexpression in HCC.

Genes usually interact with other genes, thereby exerting their biological roles [21]. To have a better understand of UBE2I action mechanism, a UBE2I-involved PPI network was established by STRING database. Enrichment analysis for the genes in this network revealed that they were significantly enriched in ubiquitin mediated proteolysis and SUMOylation pathways. These pathways have been well documented to be correlated with cancer progression [33, 34]. Correlation analysis revealed that UBE2I was significantly positively correlated with the 10 other genes in UBE2I-involved regulatory network. These correlated genes may play key roles in HCC. Especially, high expression of 6 genes (NSMCE2, SAE1, UBA2, RANGAP1, SUMO1 and SUMO2) indicated unfavorable OS and RFS in HCC. Guo et al. found that the expression levels of SUMO1 in human HCC cell lines and clinical HCC samples were significantly higher than that in corresponding normal controls [35]. All these findings suggest possible oncogenic roles of the 6 correlated genes in HCC.

## Conclusion

In conclusion, we comprehensively analyzed the expression and prognostic values of UBE2I in human cancers. We also found that UBE2I possessed a diagnostic value in HCC. Two possible molecular mechanisms were discovered to account for UBE2I overexpression in HCC. Exploration and study of UBE2I-related PPI regulatory network provided important clues for developing novel therapeutic targets in HCC. These findings need to be further confirmed in the future.

## Data Availability

Contact with correspondence author for any data and material in this study.
